# Supplementary material for: Protist–Lactic Acid Bacteria Co-Culture as a Strategy to Bioaccumulate Polyunsaturated Fatty Acids in the Protist Aurantiochytrium sp. T66
Source: Mar Drugs. 2023 Feb 23;21(3):142. doi: 10.3390/md21030142 (PMC10051163; doi:10.3390/md21030142)
Supplement: Supplementary file 1 [file marinedrugs-21-00142-s001.zip › marinedrugs-2213787-supplementary.pdf]

Supplementary material

Table. S1 – Total variance explained of PCA analysis (Figure 3)

Component	96 h			144 h		
	Eigenvalue total	% of variance	% cumulative	Eigenvalue total	% of variance	% cumulative
1	4.9	44.8	44.8	4.9	44.6	44.6
2	3.7	33.5	78.3	3.1	28.2	72.8
3	1.4	12.8	91.1	1.4	12.6	85.5

Table S2. Total lipids, total SAFA and Total MUfa content (mg g-1 biomass)

T	Item	**Total Lipids (mg·g^{-1} biomass)**		**Total SAFA (mg·g^{-1} biomass)**		**Total MUFA (mg·g^{-1} biomass)**	
		96 h	144 h	96 h	144 h	96 h	144 h
Control	Au	202.4 ± 6.4	245.7 ± 7.9	189.1 ± 5.3	234.0 ± 7.8	3.3 ± 1.6	2.8 ± 0.4
0 h	B6	**35.0 ± 10.0** *	**30.4 ± 5.6** *	**12.9 ± 5.1** *	**9.8 ± 2.8** *	**21.2 ± 1.4** *	**13.8 ± 3.1** *
	K4.5	**74.4 ± 8.0** *	**44.9 ± 2.8** *	**30.0 ± 4.8** *	**20.5 ± 2.0** *	**15.7 ± 0.8** *	**12.8 ± 1.6** *
	K6.10	**67.6 ± 3.9** *	**54.5 ± 13.0** *	**31.2 ± 1.9** *	**25.3 ± 3.1** *	**17.7 ± 1.9** *	8.2 ± 1.5
24 h	B6	**86.0 ± 11.7** *	**65.6 ± 6.2** *	**50.2 ± 10.8** *	**42.7 ± 7.4** *	12.9 ± 1.8	9.4 ± 3.0
	K4.5	**68.4 ± 4.7** *	**35.9 ± 1.3** *	**32.1 ± 1.4** *	**14.9 ± 1.1** *	14.0 ± 0.6	**12.5 ± 2.2** *
	K6.10	**79.1 ± 9.4** *	**54.4 ± 15.0** *	**41.1 ± 5.5** *	**28.9 ± 9.5** *	12.0 ± 1.2	**11.5 ± 3.4** *
72 h	B6	**91.4 ± 17.4** *	**48.3 ± 2.5** *	**54.0 ± 12.2** *	**27.8 ± 7.1** *	11.8 ± 2.5	**12.3 ± 1.8** *
	K4.5	**115.6 ± 21.9** *	**47.1 ± 1.9** *	**73.3 ± 16.8** *	**25.4 ± 0.7** *	12.4 ± 3.2	7.8 ± 0.4
	K6.10	**102.1 ± 14.8** *	**84.5 ± 14.7** *	**67.4 ± 10.4** *	**45.8 ± 11.6** *	12.4 ± 2.5	7.8 ± 1.5

Au: *Aurantiochytrium* sp. T66 (control); **B6**: Co-culture between *Aurantiochytrium* sp. T66 and *Azospirillum brasilensis* B6 (reference); **K4.5**: Co-culture between *Aurantiochytrium* sp. T66 and *Schleiferilactobacillus harbinensis* K4.5; **K6.10**: Co–culture between *Aurantiochytrium* sp. T66 and *Lentilactobacillus kefiri* K6.10; **T:** Inoculation time of the bacteria in the culture of *Aurantiochytrium* sp. T66. * in bold represents the significant differences with respect to the control (Au) with p–values < 0.05. Values indicate means ± SD.

Table S3. Lipid content (%) and fatty acid profile (% of total fatty acid) co-cultivation assay at 96 and 144 h after initiation of culture.

		96 h										144 h									
			T1			T2			T3				T1			T2			T3		
		Au	B6	K4.5	K6.10	B6	K4.5	K6.10	B6	K4.5	K6.10	Au	B6	K4.5	K6.10	B6	K4.5	K6.10	B6	K4.5	K6.10
	% Total lipids	20,24 ± 0,64	3,50 ± 1,00	6,73 ± 0,99	6,14 ± 0,93	8,60 ± 1,17	8,06 ± 1,48	7,63 ± 1,32	8,74 ± 2,40	10,32 ± 3,31	9,36 ± 0,87	24,90 ± 1,09	3,23 ± 1,30	4,84 ± 1,50	5,72 ± 2,01	7,25 ± 2,24	3,16 ± 0,72	7,70 ± 3,19	6,65 ± 3,37	4,71 ± 0,19	9,15 ± 2,88
C4:0	Butanoic acid	0,00 ± 0,00	0,00 ± 0,00	0,00 ± 0,00	0,00 ± 0,00	0,00 ± 0,00	0,01 ± 0,02	0,11 ± 0,08	0,00 ± 0,00	0,00 ± 0,00	0,00 ± 0,00	0,00 ± 0,00	0,00 ± 0,00	0,00 ± 0,00	0,00 ± 0,00	0,00 ± 0,00	0,00 ± 0,00	0,00 ± 0,00	0,00 ± 0,00	0,00 ± 0,00	0,00 ± 0,00
C6:0	Caproic acid	0,00 ± 0,00	0,00 ± 0,00	0,00 ± 0,00	0,00 ± 0,00	0,00 ± 0,00	0,00 ± 0,00	0,00 ± 0,00	0,00 ± 0,00	0,00 ± 0,00	0,00 ± 0,00	0,02 ± 0,01	0,00 ± 0,00	0,00 ± 0,00	0,00 ± 0,00	0,00 ± 0,00	0,00 ± 0,00	0,00 ± 0,00	0,00 ± 0,00	0,00 ± 0,00	0,00 ± 0,00
C8:0	Caprylic acid	0,00 ± 0,00	0,00 ± 0,00	0,00 ± 0,00	0,00 ± 0,00	0,00 ± 0,00	0,00 ± 0,00	0,00 ± 0,00	0,00 ± 0,00	0,00 ± 0,00	0,00 ± 0,00	0,02 ± 0,01	0,00 ± 0,00	0,00 ± 0,00	0,00 ± 0,00	0,00 ± 0,00	0,00 ± 0,00	0,00 ± 0,00	0,00 ± 0,00	0,00 ± 0,00	0,00 ± 0,00
C10:0	Capric Acid	0,00 ± 0,00	0,10 ± 0,17	0,00 ± 0,00	0,00 ± 0,00	0,00 ± 0,00	0,01 ± 0,02	0,00 ± 0,00	0,00 ± 0,00	0,00 ± 0,00	0,00 ± 0,00	0,03 ± 0,00	0,26 ± 0,24	0,00 ± 0,00	0,02 ± 0,04	0,00 ± 0,00	0,02 ± 0,03	0,01 ± 0,01	0,00 ± 0,00	0,06 ± 0,06	0,00 ± 0,01
C11:0	Undecanoic acid	0,03 ± 0,04	0,07 ± 0,12	0,00 ± 0,00	0,00 ± 0,00	0,00 ± 0,00	0,01 ± 0,01	0,00 ± 0,00	0,00 ± 0,00	0,03 ± 0,05	0,00 ± 0,00	0,12 ± 0,01	0,00 ± 0,00	0,00 ± 0,00	0,00 ± 0,00	0,00 ± 0,00	0,00 ± 0,00	0,01 ± 0,02	0,00 ± 0,00	0,00 ± 0,00	0,02 ± 0,02
C12:0	Lauric Acid	0,36 ± 0,33	0,76 ± 0,36	0,12 ± 0,06	0,51 ± 0,43	0,21 ± 0,09	0,18 ± 0,05	0,18 ± 0,04	0,23 ± 0,01	0,41 ± 0,18	0,25 ± 0,08	0,29 ± 0,03	2,23 ± 1,43	0,56 ± 0,36	0,32 ± 0,23	0,39 ± 0,21	0,85 ± 0,32	0,68 ± 0,44	0,73 ± 0,32	0,43 ± 0,14	0,34 ± 0,08
C13:0	Tridecanoic acid	0,76 ± 0,26	0,12 ± 0,20	0,10 ± 0,05	0,12 ± 0,02	0,43 ± 0,34	0,28 ± 0,19	0,66 ± 0,47	0,47 ± 0,31	0,51 ± 0,12	0,76 ± 0,21	2,28 ± 0,07	0,00 ± 0,00	0,24 ± 0,10	0,06 ± 0,11	0,44 ± 0,12	0,00 ± 0,00	0,39 ± 0,31	0,25 ± 0,19	0,40 ± 0,04	0,64 ± 0,46
C14:0	Myristic acid	8,48 ± 0,55	2,15 ± 0,60	2,59 ± 0,38	3,10 ± 0,58	4,73 ± 0,19	3,82 ± 0,90	4,96 ± 0,80	6,74 ± 1,08	4,52 ± 0,18	5,77 ± 0,71	7,11 ± 0,29	2,15 ± 0,84	3,23 ± 0,26	2,69 ± 1,41	5,44 ± 1,40	2,48 ± 0,57	4,78 ± 0,76	4,53 ± 0,40	5,79 ± 0,64	3,77 ± 0,16
C15:0	Pentadecanoic Acid	27,02 ± 1,25	2,38 ± 0,42	5,69 ± 1,09	5,92 ± 0,43	11,94 ± 7,08	8,87 ± 3,15	11,22 ± 1,01	9,24 ± 1,53	10,86 ± 0,99	16,43 ± 1,51	47,90 ± 1,28	3,52 ± 1,77	8,09 ± 1,91	5,83 ± 0,97	13,17 ± 5,14	4,11 ± 0,56	6,73 ± 0,34	8,69 ± 6,28	10,11 ± 0,96	10,75 ± 0,90
C16:0	palmitic acid	46,08 ± 2,41	19,10 ± 1,64	24,55 ± 0,58	26,55 ± 0,74	32,72 ± 4,29	26,59 ± 0,41	27,17 ± 1,39	33,65 ± 1,90	33,22 ± 0,67	32,35 ± 0,57	26,16 ± 1,91	15,70 ± 3,35	25,87 ± 3,70	29,46 ± 2,77	36,40 ± 4,05	25,62 ± 3,10	29,91 ± 0,83	33,96 ± 2,71	29,13 ± 1,56	26,38 ± 0,91
C17:0	heptadecanoic acid	7,81 ± 0,29	1,32 ± 0,27	2,84 ± 0,36	3,14 ± 0,10	4,18 ± 1,94	3,58 ± 0,73	3,68 ± 0,46	3,63 ± 0,55	10,24 ± 1,20	7,04 ± 1,62	9,44 ± 3,17	1,83 ± 0,52	3,51 ± 0,42	3,19 ± 0,08	5,09 ± 2,80	2,90 ± 0,03	5,56 ± 3,60	4,24 ± 2,64	3,36 ± 0,92	6,20 ± 1,20
C18:0	Stearic acid	2,50 ± 0,08	8,89 ± 0,54	3,05 ± 0,33	5,65 ± 2,22	2,52 ± 0,46	2,52 ± 0,52	2,66 ± 0,31	2,76 ± 0,40	1,98 ± 0,36	2,31 ± 0,32	1,47 ± 0,32	6,12 ± 2,98	2,87 ± 0,21	4,19 ± 1,18	2,93 ± 0,29	4,57 ± 0,50	3,11 ± 0,63	3,36 ± 0,38	3,01 ± 0,21	3,69 ± 2,39
C20:0	Arachidic Acid	0,12 ± 0,00	0,77 ± 0,25	0,07 ± 0,08	0,17 ± 0,16	0,21 ± 0,18	0,05 ± 0,04	0,07 ± 0,03	0,08 ± 0,09	0,03 ± 0,04	0,04 ± 0,04	0,09 ± 0,02	0,29 ± 0,10	0,18 ± 0,15	0,32 ± 0,02	0,16 ± 0,04	0,19 ± 0,17	0,18 ± 0,12	0,19 ± 0,16	0,14 ± 0,15	0,04 ± 0,03
C21:0	Heneicosanoic acid	0,00 ± 0,00	0,00 ± 0,00	0,12 ± 0,03	0,09 ± 0,09	0,07 ± 0,04	0,07 ± 0,04	0,13 ± 0,07	0,19 ± 0,13	0,13 ± 0,05	0,07 ± 0,06	0,07 ± 0,01	0,00 ± 0,00	0,09 ± 0,08	0,00 ± 0,00	0,04 ± 0,03	0,00 ± 0,00	0,12 ± 0,07	0,10 ± 0,09	0,16 ± 0,09	0,18 ± 0,07
C22:0	Behenic acid	0,10 ± 0,04	0,13 ± 0,23	0,31 ± 0,04	0,27 ± 0,14	0,29 ± 0,07	0,28 ± 0,04	0,25 ± 0,03	0,44 ± 0,13	0,10 ± 0,17	0,00 ± 0,00	0,06 ± 0,03	0,00 ± 0,00	0,25 ± 0,08	0,31 ± 0,02	0,21 ± 0,02	0,23 ± 0,17	0,32 ± 0,04	0,31 ± 0,07	0,37 ± 0,08	0,46 ± 0,04
C23:0	Tricosanoic Acid	0,05 ± 0,08	0,00 ± 0,00	0,57 ± 0,12	0,45 ± 0,22	0,61 ± 0,24	0,57 ± 0,28	0,45 ± 0,31	1,16 ± 0,42	0,58 ± 0,01	0,70 ± 0,12	0,05 ± 0,03	0,21 ± 0,11	0,48 ± 0,13	0,53 ± 0,05	0,46 ± 0,15	0,52 ± 0,14	0,64 ± 0,14	0,69 ± 0,08	0,67 ± 0,14	0,88 ± 0,07
C24:0	Lignoceric Acid	0,15 ± 0,09	0,43 ± 0,15	0,14 ± 0,02	0,23 ± 0,04	0,11 ± 0,03	0,28 ± 0,04	0,37 ± 0,16	0,29 ± 0,06	0,51 ± 0,17	0,26 ± 0,04	0,14 ± 0,02	0,68 ± 0,45	0,11 ± 0,06	0,28 ± 0,01	0,18 ± 0,04	0,29 ± 0,20	0,17 ± 0,09	0,21 ± 0,11	0,30 ± 0,05	0,35 ± 0,16
C14:1	Myristoleic acid	0,04 ± 0,06	25,82 ± 0,86	12,72 ± 1,05	15,88 ± 1,47	9,06 ± 1,92	9,82 ± 2,79	9,46 ± 1,04	6,22 ± 0,43	5,61 ± 1,51	5,80 ± 1,10	0,06 ± 0,01	33,24 ± ####	17,86 ± 2,46	10,44 ± 1,54	8,26 ± 3,75	21,26 ± 3,40	12,49 ± 0,69	11,87 ± 7,37	9,33 ± 0,64	5,51 ± 0,35
C15:1	Cis-10-Pentadecenoic acid	0,05 ± 0,07	5,78 ± 1,76	2,68 ± 0,57	3,29 ± 0,69	1,12 ± 0,10	1,94 ± 0,99	1,15 ± 0,07	1,30 ± 0,05	1,11 ± 0,17	1,20 ± 0,36	0,17 ± 0,03	6,16 ± 2,15	4,16 ± 0,99	3,70 ± 2,32	1,62 ± 0,77	4,97 ± 0,56	2,57 ± 0,11	2,31 ± 1,71	2,02 ± 0,80	0,61 ± 0,07
C16:1	Palmitoleic acid	0,23 ± 0,15	9,65 ± 3,22	3,48 ± 0,42	4,42 ± 0,40	2,29 ± 0,60	2,94 ± 0,83	2,88 ± 0,48	2,01 ± 0,56	2,34 ± 0,89	1,94 ± 0,22	0,21 ± 0,02	9,42 ± 2,24	4,29 ± 1,18	3,89 ± 2,16	1,95 ± 1,22	5,27 ± 0,67	3,60 ± 0,24	3,26 ± 2,28	2,32 ± 0,46	1,54 ± 0,12
C17:1	Cis-10-Heptadecenoic acid	0,09 ± 0,13	1,08 ± 0,32	0,52 ± 0,10	0,57 ± 0,13	0,33 ± 0,07	0,46 ± 0,03	0,28 ± 0,00	0,52 ± 0,13	0,57 ± 0,36	0,55 ± 0,09	0,28 ± 0,04	0,90 ± 0,05	0,59 ± 0,15	0,65 ± 0,22	0,33 ± 0,16	0,87 ± 0,11	0,59 ± 0,08	0,50 ± 0,18	0,57 ± 0,11	0,40 ± 0,10
C18:1n9t	Elaidic Acid	0,44 ± 0,62	1,83 ± 2,26	0,18 ± 0,01	0,48 ± 0,32	0,27 ± 0,04	0,47 ± 0,20	0,29 ± 0,14	0,82 ± 0,73	0,46 ± 0,23	0,83 ± 0,26	0,09 ± 0,04	1,87 ± 1,42	0,24 ± 0,14	0,37 ± 0,01	1,02 ± 0,90	0,52 ± 0,12	0,31 ± 0,09	0,41 ± 0,08	0,95 ± 0,79	0,22 ± 0,05
C18:1n9c	Oleic acid	0,38 ± 0,22	7,13 ± 2,69	0,94 ± 0,27	1,19 ± 0,52	1,53 ± 0,55	1,04 ± 0,17	0,72 ± 0,02	1,32 ± 0,53	1,17 ± 0,55	1,32 ± 0,59	0,16 ± 0,05	3,07 ± 0,02	0,95 ± 0,34	1,21 ± 0,05	1,17 ± 0,30	1,58 ± 1,20	1,11 ± 0,17	1,23 ± 0,27	1,16 ± 0,19	0,59 ± 0,18
C20:1n9	Cis-11-Eicosenoic Acid	0,14 ± 0,19	0,00 ± 0,00	0,00 ± 0,00	0,00 ± 0,00	0,04 ± 0,00	0,02 ± 0,03	0,05 ± 0,05	0,03 ± 0,05	0,03 ± 0,04	0,03 ± 0,05	0,02 ± 0,01	1,07 ± 0,69	0,02 ± 0,03	0,00 ± 0,00	0,00 ± 0,00	0,00 ± 0,00	0,00 ± 0,00	0,00 ± 0,00	0,04 ± 0,07	0,03 ± 0,04
C22:1n9	Erucic acid	0,19 ± 0,15	1,18 ± 0,36	0,42 ± 0,11	0,13 ± 0,13	0,36 ± 0,03	0,34 ± 0,09	0,21 ± 0,19	0,39 ± 0,14	0,33 ± 0,14	0,19 ± 0,04	0,09 ± 0,03	0,52 ± 0,33	0,31 ± 0,05	0,32 ± 0,03	0,07 ± 0,07	0,02 ± 0,04	0,08 ± 0,09	0,00 ± 0,00	0,05 ± 0,08	0,02 ± 0,02
C24:1n9	Nervonic acid	0,10 ± 0,05	0,18 ± 0,16	0,33 ± 0,11	0,25 ± 0,07	0,19 ± 0,05	0,15 ± 0,13	0,13 ± 0,08	0,17 ± 0,05	0,17 ± 0,03	0,21 ± 0,08	0,06 ± 0,04	0,30 ± 0,13	0,19 ± 0,00	0,26 ± 0,03	0,13 ± 0,06	0,35 ± 0,27	0,24 ± 0,03	0,20 ± 0,08	0,17 ± 0,04	0,34 ± 0,09
C18:2n6t	Linolelaidic Acid	0,23 ± 0,02	0,83 ± 0,45	0,17 ± 0,02	0,21 ± 0,02	0,28 ± 0,10	0,22 ± 0,05	0,29 ± 0,16	0,24 ± 0,17	0,42 ± 0,44	0,27 ± 0,07	0,54 ± 0,02	0,79 ± 0,07	0,16 ± 0,02	0,26 ± 0,06	0,21 ± 0,08	0,30 ± 0,15	0,29 ± 0,16	0,27 ± 0,12	0,27 ± 0,11	0,49 ± 0,36
C18:2n6c	Linoleic acid	0,14 ± 0,01	1,01 ± 0,40	0,20 ± 0,00	0,26 ± 0,13	0,29 ± 0,17	0,26 ± 0,13	0,19 ± 0,06	0,21 ± 0,04	0,24 ± 0,03	0,10 ± 0,02	0,06 ± 0,02	0,47 ± 0,22	0,20 ± 0,05	0,24 ± 0,02	0,14 ± 0,08	0,36 ± 0,13	0,17 ± 0,03	0,20 ± 0,08	0,18 ± 0,03	0,16 ± 0,04
C18:3n3	Linolenic Acid	0,00 ± 0,00	0,10 ± 0,17	0,05 ± 0,01	0,04 ± 0,07	0,07 ± 0,01	0,06 ± 0,03	0,07 ± 0,01	0,08 ± 0,03	0,16 ± 0,21	0,03 ± 0,05	0,01 ± 0,01	0,00 ± 0,00	0,02 ± 0,03	0,00 ± 0,00	0,00 ± 0,00	0,00 ± 0,00	0,03 ± 0,05	0,03 ± 0,05	0,06 ± 0,05	0,08 ± 0,03
C18:3n6	g-linolenic acid	0,06 ± 0,08	0,00 ± 0,00	0,26 ± 0,09	0,19 ± 0,20	0,13 ± 0,16	0,25 ± 0,02	0,27 ± 0,03	0,34 ± 0,06	0,31 ± 0,08	0,22 ± 0,04	0,01 ± 0,01	0,55 ± 0,54	0,09 ± 0,15	0,00 ± 0,00	0,06 ± 0,06	0,03 ± 0,05	0,10 ± 0,14	0,12 ± 0,21	0,32 ± 0,13	0,35 ± 0,02
C20:2	Eicosadienoic Acid	0,30 ± 0,42	1,34 ± 2,33	0,01 ± 0,02	0,17 ± 0,14	0,07 ± 0,08	0,12 ± 0,16	0,13 ± 0,10	0,08 ± 0,09	0,30 ± 0,20	0,25 ± 0,11	0,04 ± 0,03	1,45 ± 0,81	0,18 ± 0,07	0,12 ± 0,12	0,07 ± 0,11	0,00 ± 0,00	0,01 ± 0,02	0,00 ± 0,00	0,15 ± 0,16	0,09 ± 0,08
C20:3n3	Eicosatrienoic Acid	0,00 ± 0,00	0,18 ± 0,31	0,04 ± 0,04	0,03 ± 0,06	0,03 ± 0,05	0,14 ± 0,09	0,17 ± 0,15	0,19 ± 0,12	0,15 ± 0,27	0,06 ± 0,05	0,02 ± 0,01	0,00 ± 0,00	0,00 ± 0,00	0,00 ± 0,00	0,07 ± 0,02	0,27 ± 0,40	0,09 ± 0,04	0,05 ± 0,04	0,17 ± 0,19	0,11 ± 0,05
C20:3n6	Eicosatrienoic Acid	0,00 ± 0,00	0,00 ± 0,00	0,00 ± 0,00	0,00 ± 0,00	0,09 ± 0,11	0,10 ± 0,09	0,15 ± 0,09	0,29 ± 0,15	0,14 ± 0,23	0,10 ± 0,09	0,05 ± 0,01	0,00 ± 0,00	0,00 ± 0,00	0,08 ± 0,14	0,00 ± 0,00	0,00 ± 0,00	0,09 ± 0,08	0,00 ± 0,00	0,03 ± 0,03	0,15 ± 0,06
C20:4n6	Arachidonic Acid	0,04 ± 0,05	0,00 ± 0,00	0,45 ± 0,07	0,71 ± 0,03	0,33 ± 0,01	0,25 ± 0,03	0,47 ± 0,02	0,37 ± 0,03	0,25 ± 0,05	0,26 ± 0,01	0,05 ± 0,02	0,67 ± 0,03	0,57 ± 0,12	0,64 ± 0,16	0,49 ± 0,04	0,63 ± 0,02	0,58 ± 0,03	0,55 ± 0,08	0,50 ± 0,13	0,74 ± 0,09
C20:5n3	Eicosapentaenoic Acid	0,14 ± 0,03	0,26 ± 0,23	1,57 ± 0,09	1,83 ± 0,28	1,14 ± 0,07	0,96 ± 0,09	1,54 ± 0,10	0,88 ± 0,13	0,82 ± 0,02	0,83 ± 0,08	0,15 ± 0,01	0,30 ± 0,09	1,21 ± 0,30	1,31 ± 0,17	1,38 ± 0,25	1,64 ± 0,04	1,54 ± 0,23	1,34 ± 0,17	1,05 ± 0,35	1,64 ± 0,14
C22:2	Docosadienoic acid	0,00 ± 0,00	0,00 ± 0,00	0,02 ± 0,03	0,00 ± 0,00	0,04 ± 0,01	0,06 ± 0,01	0,06 ± 0,03	0,14 ± 0,07	0,11 ± 0,12	0,00 ± 0,00	0,03 ± 0,01	0,38 ± 0,34	0,52 ± 0,07	0,56 ± 0,05	0,08 ± 0,08	0,00 ± 0,00	0,01 ± 0,01	0,00 ± 0,00	0,00 ± 0,00	0,02 ± 0,01
C22:5n6	Docosapentaenoic acid	1,18 ± 0,30	2,01 ± 0,33	7,70 ± 0,83	6,33 ± 0,29	5,45 ± 0,36	7,30 ± 0,14	6,98 ± 0,62	6,12 ± 0,70	5,25 ± 0,72	5,19 ± 0,53	0,85 ± 0,20	2,20 ± 0,88	5,58 ± 0,55	7,08 ± 0,69	4,62 ± 0,54	4,49 ± 0,29	5,79 ± 0,37	5,75 ± 0,21	7,17 ± 0,83	9,65 ± 0,93
C22:6n3	Docosahexaenoic acid	2,79 ± 0,47	5,41 ± 1,62	28,10 ± 0,62	17,76 ± 1,21	18,85 ± 1,38	25,99 ± 0,78	22,59 ± 0,71	19,30 ± 1,98	16,95 ± 2,61	14,62 ± 0,67	1,82 ± 0,36	3,66 ± 1,00	17,38 ± 1,21	21,67 ± 1,85	13,41 ± 1,63	15,66 ± 0,70	17,72 ± 2,74	14,65 ± 0,49	19,57 ± 0,34	23,53 ± 4,10
	∑ SAFA	93,46 ± 0,35	36,21 ± 4,05	40,17 ± 2,28	46,21 ± 1,42	58,03 ± 4,84	47,12 ± 3,90	51,91 ± 1,51	58,88 ± 2,44	63,11 ± 2,88	65,99 ± 1,98	95,25 ± 0,48	32,99 ± 10,5	45,49 ± 2,10	47,20 ± 5,42	64,91 ± 7,29	41,77 ± 4,61	52,60 ± 2,50	57,26 ± 11,7	53,92 ± 0,69	53,72 ± 4,46
	∑ MUFA	1,66 ± 0,85	52,65 ± 4,25	21,26 ± 2,16	26,21 ± 3,00	15,19 ± 3,19	17,17 ± 4,45	15,18 ± 1,10	12,89 ± 0,24	11,79 ± 1,68	12,08 ± 1,35	1,13 ± 0,15	56,55 ± 13,6	28,59 ± 4,37	20,83 ± 6,23	14,56 ± 5,34	34,85 ± 4,71	20,99 ± 0,46	19,79 ± 11,6	16,61 ± 0,39	9,27 ± 0,56
	∑ PUFA	4,87 ± 1,20	11,14 ± 4,57	38,57 ± 0,13	27,58 ± 1,81	26,77 ± 1,65	35,71 ± 0,70	32,91 ± 0,42	28,24 ± 2,66	25,10 ± 2,21	21,93 ± 1,01	3,62 ± 0,53	10,46 ± 3,16	25,91 ± 2,28	31,97 ± 2,46	20,53 ± 2,32	23,38 ± 1,19	26,41 ± 2,93	22,95 ± 0,98	29,47 ± 0,56	37,01 ± 4,77

* The values correspond to means ± SD n=3 of each test, each replicate presents analytical duplicate.
∑SAFA: Total saturated fatty acids.
∑MUFA: Total monounsaturated fatty acids.
∑PUFA: Total polyunsaturated fatty acids.

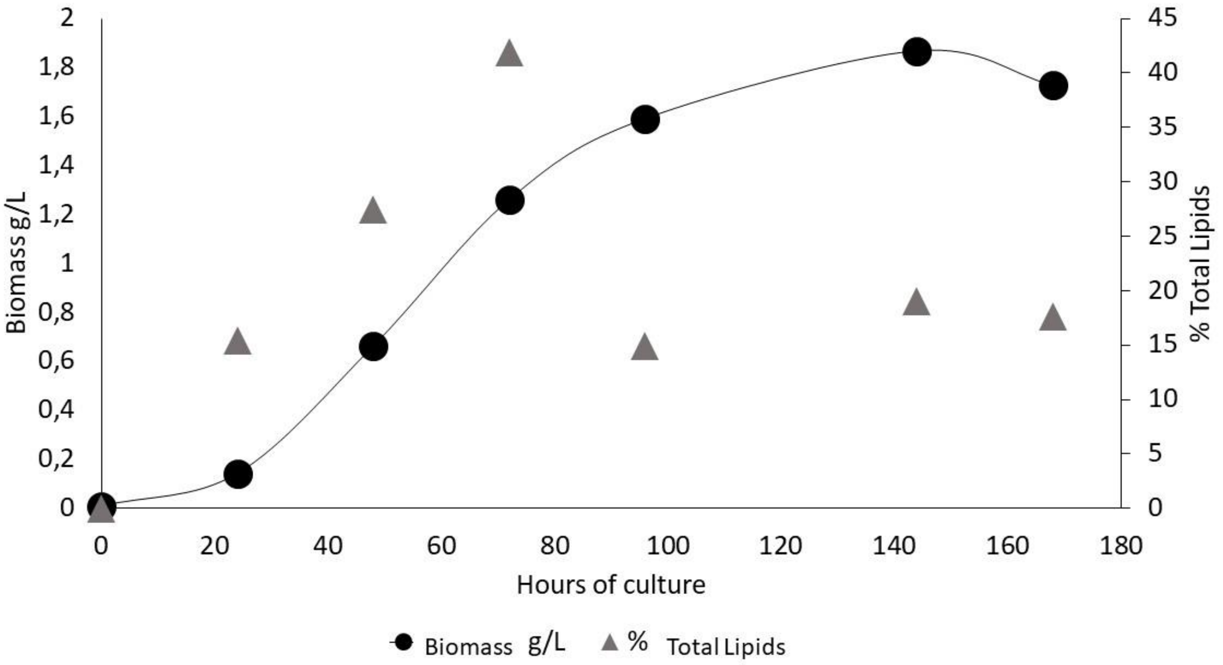

Figure S1. Growth kinetics of *Aurantiochytrium* sp. T66 in the 2673 medium and total lipids acumulation
